# NK cell education: Physiological and pathological influences

**DOI:** 10.3389/fimmu.2023.1087155

**Published:** 2023-01-20

**Authors:** Philippe Rascle, Griffin Woolley, Stephanie Jost, Cordelia Manickam, R. Keith Reeves

**Affiliations:** ^1^ Division of Innate and Comparative Immunology, Center for Human Systems Immunology, Duke University School of Medicine, Durham, NC, United States; ^2^ Department of Surgery, Duke University School of Medicine, Durham, NC, United States; ^3^ Center for Virology and Vaccine Research, Beth Israel Deaconess Medical Center, Harvard Medical School, Boston, MA, United States

**Keywords:** natural killer cell, NK cell education, NKG2A, transplant, cancer, KIR, viral infections

## Abstract

Natural killer (NK) cells represent a critical defense against viral infections and cancers. NK cells require integration of activating and inhibitory NK cell receptors to detect target cells and the balance of these NK cell inputs defines the global NK cell response. The sensitivity of the response is largely defined by interactions between self-major histocompatibility complex class I (MHC-I) molecules and specific inhibitory NK cell receptors, so-called NK cell education. Thus, NK cell education is a crucial process to generate tuned effector NK cell responses in different diseases. In this review, we discuss the relationship between NK cell education and physiologic factors (type of self-MHC-I, self-MHC-I allelic variants, variant of the self-MHC-I-binding peptides, cytokine effects and inhibitory KIR expression) underlying NK cell education profiles (effector function or metabolism). Additionally, we describe the broad-spectrum of effector educated NK cell functions on different pathologies (such as HIV-1, CMV and tumors, among others).

## Introduction

As a lymphocyte population, NK cells are essential for homeostasis and immune defense against a wide range of infectious and chronic diseases (1, 2). The heterogeneity of NK cell responses, typically either cytokine-producing or cytotoxic, is crucial for immune protection (3, 4). These effector NK cell functions are managed by a balance of activating and inhibitory input signals (5, 6). Activating NK cell receptors (aNKR), such as the natural cytotoxicity receptors (including NKp46, NKp30 and NKp44), NKG2D and DNAM-1, function as the major activating receptors involved in target cell killing *via* the recognition of cell surface ligands expressing different types of stress (non-self, dangerous) signals (7). Alternately, inhibitory receptors (iNKR), such as LAG-3, TIGIT, Tim-3, NKG2A/CD94, or inhibitory KIR translate healthy (self, non-dangerous) signals to NK cells (3, 5, 6). Meanwhile, NK cells can sense the absence of MHC-I expression. Some NK cell receptors can also detect MHC-I molecules as stress signals, such as the killer-cell immunoglobulin-like receptors (KIR) and NKG2C/CD94 (3, 5–7). The threshold of NK activation, *via* the accumulation of input signals, is calibrated by the NK cell education process (8, 9, 10). NK cell education is the recognition of self-MHC class I (MHC-I) *via* inhibitory NK cell receptors, which prevents NK cell auto-reactivity and maintains tolerance to self. The calibration of this threshold is determined by the self-MHC-I environment present during the NK cell education process and is mainly mediated by KIR, including KIR2DL1 and KIR2DL2/L3 NK cell receptors (8, 9, 11). However, other inhibitory receptors for self-MHC-I, such as NKG2A/HLA-E and KIR3DL1, can also play a role in this process and NK cells educated by NKG2A/HLA-E develop different responses compared to iKIR educated NK cells (12). Additional extrinsic factors further influence responses mediated by educated NK cells. For instance, MHC-I expression can be modulated by pathogens, allowing infected cells to escape NK cell recognition (10, 13). Moreover, educated NK cells are more sensitive to variations in MHC-I expression levels on target cells compared to uneducated NK cells (8). NK cell receptor engagement can also be influenced by changes in the peptidome presented by MHC-I, thereby significantly affecting NK cell effector functions (14). These data suggest an intricate relationship between NK cell education and the cellular environment (11).

In this review, we will discuss the relationship between NK cell education and environmental influences, with a special focus on factors affecting NK cell education in physiological niches, such as self-MHC-I expression, self-antigen MHC-I presentation, or cytokine milieu. We will also review effector educated NK cell functional profiles in pathological environments, as well as their potential ability to restrict the pathology of specific diseases.

## NK cell education governed by a specific environment

In humans, NK cells express different combinations of inhibitory receptors (such as KIR and NKG2A) that recognize self-MHC-I proteins. Based on this self-recognition, NK cells can be defined as either educated or uneducated NK cells. The educated NK cells are reactive against target cells that lack or have downregulated MHC-I and co-express activating ligands. NK cells that do not express any inhibitory receptors for self-MHC-I recognition are referred to as uneducated NK cells. Uneducated NK cells are very weakly sensitive to inhibition by self-MHC class I variations, requiring very high activation signals to become reactive. Thus, without strong stimulation, uneducated NK cells are largely hyporesponsive (8).

The education of NK cells is typically described through four models (8). In the first model, the licensing model (or arming model), NK cells acquire high effector capabilities by the recognition of self-MHC-I ligands *via* expressed iNKR (8, 15). The second model, the disarming model, describes high effector capabilities in NK cells, which become anergic as soon as they fail at recognizing self-MHC-I (8). Thirdly, the rheostat model characterizes the relationship between NK cell education and the quantity of interactions with self-MHC-I ligands, and is therefore pertinent in both the licensing and disarming models (8, 16). Lastly, the tuning model defines the evolution of NK cell education due to fluctuations of self-MHC-I expressed in the cellular environment over time (8, 17, 18). According to these models, NK cell education seems to actively participate in NK cell functional plasticity and could be involved in modulating the effector NK cell repertoire during chronic diseases. Herein we will assess the relative importance of each in disease states.

## NK cell education in humans

In humans, KIR2DL1/2/3 and KIR3DL1 receptors play an important role in the NK cell education process. First, KIR3DL1 is expressed at varying levels on NK cells and binds to HLA-B and HLA-A bearing Bw4 allotypes, such as HLA-A*24:02, -A*32:01, -A*23:01, and HLA-Bw4 ligands. The HLA-B alleles are divided into Bw6 or Bw4 epitopes, with further division of HLA-Bw4 into HLA-Bw4-80I or HLA-Bw4-80T subtypes based on a dimorphism (isoleucine, I vs threonine, T) at position 80 (Bw4*80I and Bw4*80T). The HLA-Bw4*80I genotype is associated with both low and high KIR3DL1 expression levels on educated NK cells, and presents a higher expression density and ligand-binding affinity than HLA-Bw4*80T (8, 19–22). While HLA-Bw4 facilitates KIR3DL1 binding, HLA-Bw6 does not interact with any KIR3DL1 (23–25). In individuals homozygous for HLA-Bw6, NK cells are not sufficiently educated through KIR3DL1 receptors (8, 19, 20). Besides HLA-Bw4, KIR3DL1 can be educated by HLA-A*24 and HLA-A*32 allotypes (26). KIR3DL1 allotypes present distinct hierarchies of HLA-Bw4 recognition that are independent of HLA-Bw4*80I/T variants (27). Indeed, KIR3DL1*005 displays wider, high-binding preferences to HLA-I ligand recognition than KIR3DL1*001, which itself has higher binding preferences compared to KIR3DL1*015 (27).

KIR2DL1/2/3 can also educate NK cells, but through engagement with HLA-C variants 1 or 2 and two HLA-B allotypes (HLA-B*46:01 and HLA-B*73:01) (19). KIR2DL1 binds HLA-C2 with high affinity, while KIR2DL2/3 recognizes HLA-C1 (8, 19). KIR2DL1/2/3 and HLA-C interactions are dependent on the HLA-C allotypes. For example, KIR2DL1 molecules recognize distinct HLA-C2 allotypes with different avidities. KIR2DL1 expresses the highest affinity for HLA-C*15:02 and the lowest for HLA-C*04:01. Similarly, KIR2DL2/3 and HLA-C1 interactions exhibit different avidities, where KIR2DL2/3 presents the highest affinity for HLA-C*03:03 and the lowest for HLA-C*01:02 (28). The efficiency of NK cell education by KIR2DL1/2/3 is also dependent on different mutations on iKIR. For instance, the (35E) variant of KIR2DL2/L3 presents better affinity in HLA-C1 engagement than its (35Q) variant. Meanwhile, the KIR2DL1 (245R) variant presents better inhibition than its (245C) variant in terms of HLA-C2 engagement (8, 19, 29, 30). Interestingly, the activating KIR2DS1^+^ NK cell receptor, known to interact exclusively with HLA-C2 molecules, has been associated with hyporesponsive responses by NK cells from HLA-C2+ donors compared to NK cells from HLA-C1+ donors upon *in vitro* stimulation with HLA-deficient target cells (31). In this stimulation, KIR2DS1^+^ NK cells from HLA-C2^+^ donors displayed decreased NK cell degranulation in comparison with KIR-negative NK cells. Moreover, NK cells co-expressing KIR2DS1 and NKG2A were less responsive than NKG2A^+^ NK cells in HLA-C2^+^ donors. Thus, KIR2DS1 can represent a complementary mechanism to the effector function calibration in NK cell education (30–32). The efficiency of NK cell education is also dependent upon the peptide being presented. KIR2DL2/3 and KIR2DL1 are characterized by a distinctive level of detection for HLA-C peptide presentation, with a greater sensitivity for KIR2DL2/3 than KIR2DL1 and with a critical effect of amino acids on position 7 or 8 of the HLA-C-binding peptide on this recognition (28). For instance, the metalloproteinase 1–derived peptide (VAPWNSFAL) and its variant (VAPWNSDAL) bind to HLA-C1 with similar affinities (33). However, while VAPWNSFAL is a stronger inhibitor of KIR2DL3^+^ NK cells, VAPWNSDAL abrogates inhibitory signaling in NK cells by disrupting the clustering of KIR2DL3 in NK cell synapses (34). KIR3DL1 engagement can also be influenced by self-peptide presentation (14, 27). Indeed, variations of the self-peptide LSSPVTKSF (14, 35) in position 8 for glutamic acid or leucine substitutions induce a dramatic loss of the KIR3DL1 binding to HLA-B*57 (14). Altogether, the efficiency of KIR education depends on different mechanisms such as the nature of peptide presented, KIR variants, and MHC-I variants.

NK cell education does not exclusively rely on KIR, but is also calibrated by NKG2A/HLA-E engagement (8, 36). First, different HLA-E alleles display distinct levels of cell surface stabilization. It has been shown that the 107A variant (HLA-E*01:01) is less stable on the surface of cells than the 107G variant (HLA-E01:03) (37). HLA-E expression also relies on the sequence of the peptide that is presented. In healthy cells, HLA-E usually binds a signal peptide derived from the leader sequence of HLA-A, -B, and -C (37, 38). The amino acids at positions 2 and 9 are crucial to determine peptide-binding in the HLA-E pocket. While HLA-A and -C do not present any variants for this position, HLA-B displays two variants, Methionine (M) or Threonine (T), on residue -21 that correspond with position 2 on the HLA-B-derived peptide that binds HLA-E (39). HLA-E expression at the cell surface is less stable with the HLA-B (-21T) genotype than with the HLA-B (-21M) genotype. Accordingly, the HLA-B (-21M) genotype favors education of CD94/NKG2A^+^ NK cells compared to the HLA-B (-21T) genotype due to differences in the engagement of HLA-E (39).

Recently, an *in vitro* study detailed that LIR-1 (LILRB1) receptors can mediate co‐education with educated KIRs^+^ (KIR2DL1, KIR2DL2/3, and KIR3DL1) NK cells and show higher responsiveness to K562 cells than educated KIRs^+^ NK cells. In an anti-tumoral context, LILRB1^+^ NK cells express high ADCC capacities compared the other KIR and NKG2A educated NK subsets (40). NK cell education has been suggested to also occur through HLA class I-independent inhibitory interactions. For instance, in patients with X-linked lymphoproliferative disease 1 (XLP1), 2B4 and NTB-A display inhibitory signals instead of activating ones due to impaired functionality of the signaling lymphocyte activation molecule (SLAM)-associated protein (SAP). In contrast to healthy donors, XLP1 patients produce a portion of NK cells which lack iNKR for self-HLA class I molecules, are functional and mediate responses against cells lacking ligands for 2B4 or NTB-A, including autologous antigen presenting cells. Therefore, this alternate education mechanism may result in autoreactivity and, in XLP1 patients, worsen the immunodeficiency (41, 42).

Finally, studies in mice have demonstrated that NK cell education can also be led by TIGIT inhibitory receptors (43, 44). Indeed, TIGIT^+^ NK cells in CD155-deficient mice demonstrate a functional impairment (degranulation and IFNγ production). On the other hand, TIGIT deficiency induces a decrease in the NK cell response during CD155^-^ target cell stimulation. These studies in mouse models reported that the CD155 ligand supports TIGIT^+^ educated NK cells in parallel and independently of the self-MHC-I-dependent NK cell education process (44). CD226 expression is correlated with iNKR for self-MHC-I, but the absence of CD226 expression does not abrogate the missing self-killing. Thus, CD226 exhibit a close association with NK cell education but do not seem to be involved in NK cell education directly (45). SLAM family receptors (SFR) can support NK cell education despite the fact that SFR are activating NK cell receptors (45, 46). The chronic engagement of SFR mediates a desensitization of NK cell responsiveness *via* hematopoietic cell recognition. SFR deficiency affects particularly the functional acquisition of unlicensed (Ly49C−Ly49I−NKG2A−) NK cells (46).

As it is impacted by genetic or peptidome environmental changes, the NK cell education repertoire displays some specificity according to tissue (47, 48). The distinction between conventional NK cells and tissue-resident NK cells regarding NKG2A and KIR expression has been reported previously in humans (2). NK cells from blood and bone marrow demonstrated a similar major proportion of KIRs^+^ (KIR3DL1; KIR2DL1,2,3/DS1,2,5) and NKG2A^+^ NK cells with some NK cell subsets being KIRs^+^ or NKG2A^+^ only. The spleen, gut, and lymph nodes display an enrichment in KIRs^-^ NK cells while the lung presents a majority of KIRs^+^ NKG2A^+^ NK cells (2). NK cells from the liver express less iKIR (KIR2DL1 and KIR3DL1) than peripheral blood NK (pbNK) cells but show comparable levels of NKG2A. Liver NK cells also have less capacity to lyse MHC-deficient K562 and 721.221 cell lines compared to pbNK cells (49). While decidual NK (dNK) cells have a higher expression of KIR2DL1, KIR2DL3, and NKG2A than pbNK cells, pbNK cells are more strongly educated, displaying a stronger degranulation (CD107a) response to K562 cells (50). However, pbNK cells co-expressing KIR2DL1/NKG2A/KIR3DL1/KIR2DL3/LILRB1 show increased education in comparison to pbNK cells expressing KIR2DL1 and/or NKG2A. Meanwhile, dNK cells expressing KIR2DL1 and/or NKG2A were more educated than dNK cells co-expressing KIR2DL1/NKG2A/KIR3DL1/KIR2DL3/LILRB1 (50). Overall, the NK cell education repertoire displays variation and specificity according to the source tissue.

The differences in educated NK cell repertoires among diverse tissues could be due to variations in environmental factors (51). The environment can be heavily influenced by soluble factors that cause changes within immune cells (52, 53). Dendritic cells (DC) secrete several cytokines (IL- 2, IL- 12p70, IL- 15, IL- 18, IFN-α, and IFN-β) to activate NK cells (54). Lipopolysaccharides (LPS) and poly(I:C)-stimulated DC secrete IL- 12p70 (55), which induces the expression of NKG2A on hyporesponsive unlicensed NKG2A^-^ NK cells. This NK cell population then exhibits NKG2A-induced elevated CD107a^+^ and IFN-γ^+^ responses to MHC-I-negative 721.221 cell line stimulation (55). Furthermore, IL- 2 and IL- 15 play an important role in NK cell survival and function (56). These cytokines have been shown to induce NKG2A and KIR3DL1 expression on NKG2A^-^/KIR3DL1^-^ NK cells. Notably, exposure to a low dose of IL- 15 resulted in the restoration of self-KIR educated NK cell cytotoxicity and IFN-γ production during post-allogeneic hematopoietic cell transplantation (57). IL- 2 confers cytotoxic competence against K562 and 721.221 cell lines to NK cells that were initially NKG2A^-^/KIR3DL1^-^ and hyporesponsive. This new functional competence is acquired by KIR3DL1 expression *via* IL- 2, and these KIR3DL1^+^ NK cells display a self-tolerance competence by HLA-Bw4 recognition on modified 721.221 cells (58). The role of IL- 2 in promoting KIR3DL1 expression by decreasing hypomethylation in the CpG KIR3DL1 promoter region could contribute towards this functional change (58). As with IL- 2, another study demonstrated the capacity of ascorbic acid to elicit KIR promoter demethylation in NK cells (59). Thus, epigenetic modification could be an important factor in educated NK cell reprogramming.

**Figure 1:**
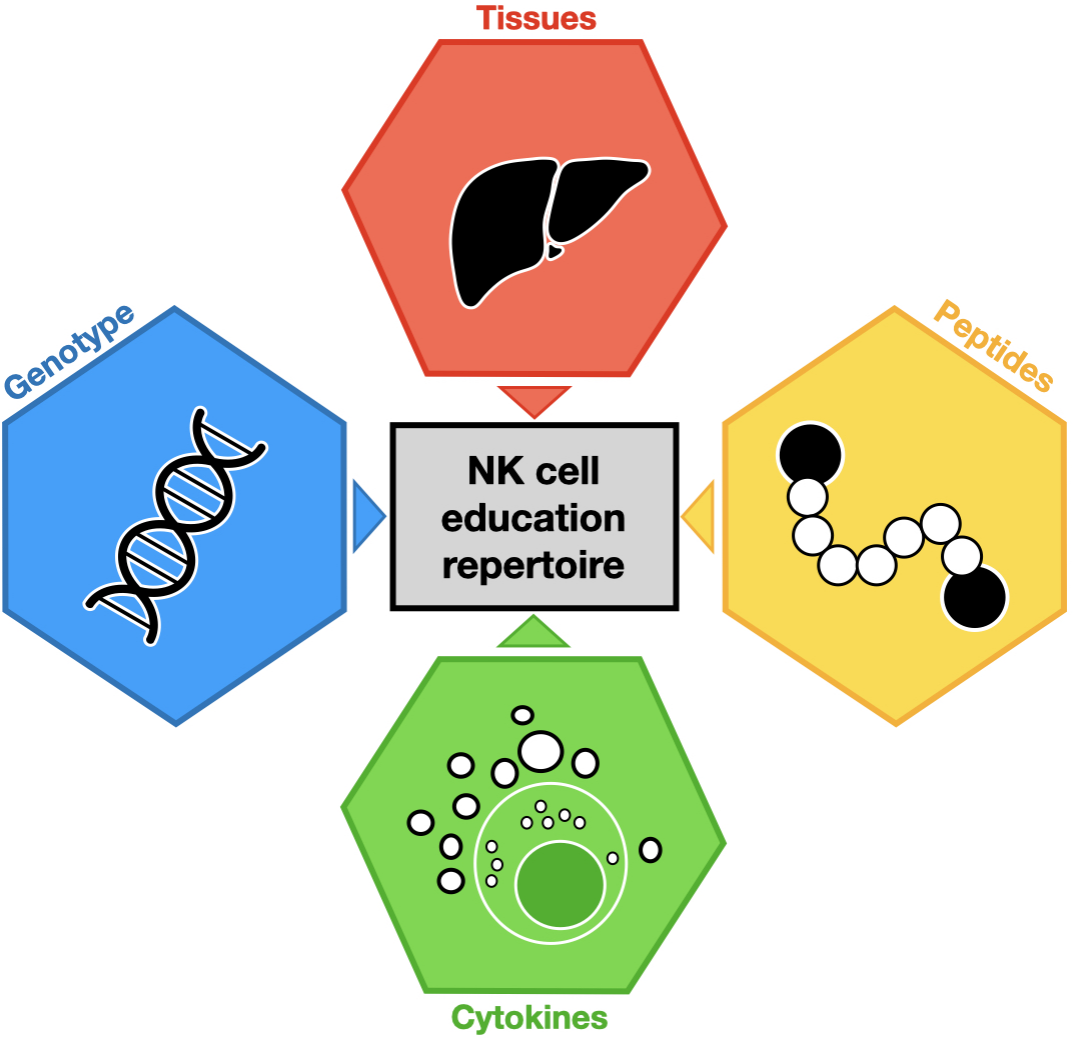
Major environment contributions to NK cell education repertoire. Each colored hexagon indicates a factor involved in NK cell education. The impact of these factors on NK cell education is symbolized by colored triangles.

NK cell metabolism can also regulate NK cell education and function. Glycosylation and mitochondrial respiration can support the proliferation and cytotoxic functions of educated NK cells (60). One study demonstrated that educated NK cells have a higher expression of glucose transporter (Glut1) than uneducated NK cells (61), resulting in a higher rate of glycolysis. These findings are subset-specific and KIR educated NK cells express more Glut1 than NKG2A educated NK cells, and the blocking of glycolysis induces a partial reduction of KIR-educated NK cell functions, contrary to NKG2A-educated NK cells (12, 61). Similarly, upon restriction of oxidative phosphorylation, KIR-educated NK cells are converted to dysfunctional cells, while the NKG2A-educated NK cells maintain their functional capacities (12, 61). These observations suggest that NKG2A-educated NK cells retain efficiency in a glucose- and oxidative phosphorylation-restricted environment compared to KIR-educated NK cells (12, 60, 61). These mechanisms describe merely a part of the broad-spectrum of NK cell education diversity within a given organism (**Figure 1**).

## NK cell education in viral infections

Viral infections generate broad changes within the host. In reaction to inflammation and viral escape by either HLA modulation or peptide changes (62, 63), educated and uneducated NK cells correspondingly exhibit variations in the magnitude of effector responses. In this section, we discuss the modulation of effector educated NK cell functions in response to different viral infections.

## Human immunodeficiency virus

In the absence of viral control by the immune system or by antiretroviral therapy, HIV infection progresses to acquired immunodeficiency syndrome (AIDS), which is characterized by opportunistic infections and tumors (64). Due to their roles in antiviral response (65–67) and notably antibody-dependent cell-mediated cytotoxicity (ADCC) (68), NK cells are major players in anti-HIV immunity. The modulation of HLA-I on target cells is an essential mechanism by which HIV escapes antiviral cell restriction. The HLA-A/-B/-C molecules are downregulated on HIV-infected cells, and some strains can also downregulate HLA-E expression (69, 70). However, HLA-E is stabilized in HIV infection by the binding of viral peptides (71). Thus, the sensitivity of NK cell activation established by education is crucial to induce an efficient effector educated NK cell response during HIV infection (72). For instance, expression of KIR3DL1*004 (in the context of self-HLA-Bw4) or that of KIR3DL1 allotypes expressed at high-density (in the context of self-HLA-Bw4*80I) have been described to delay AIDS progression (73). Indeed, HLA-Bw4*80I downregulation could increase KIR3DL1 educated NK cell sensitivity and restrict AIDS progression (21, 22). Thus, educated NK cells exhibit variation of their functionality against HIV-infected cells, according to self-HLA-I profiles.

**Figure 2:**
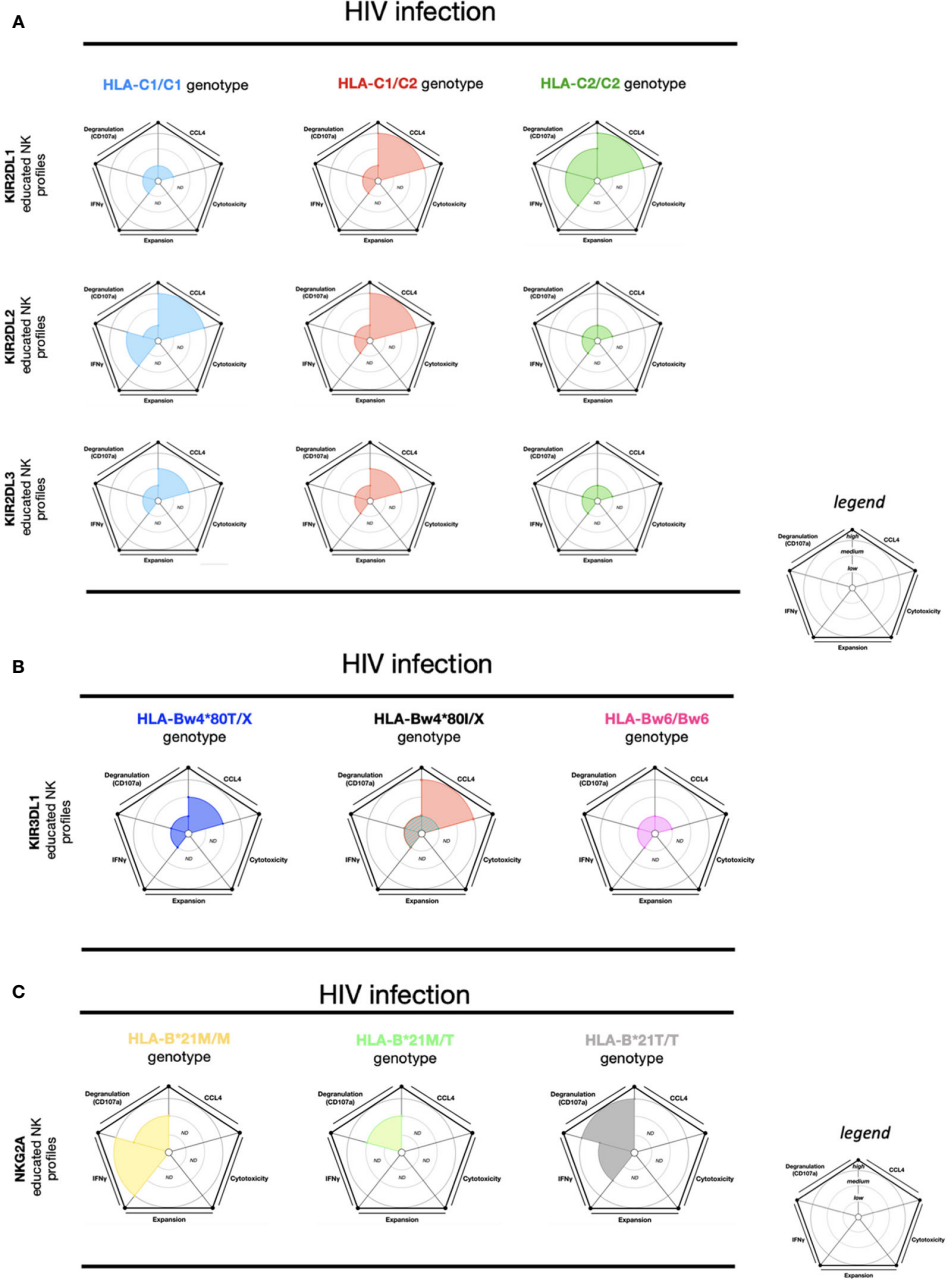
NK cell education profiles concerning to HIV infection. The different colors each represent a specific-NKR educated NK profile. Each educated NK profiles are defined by five factors (degranulation, CCL4, IFNγ, cytotoxicity, and expansion). Each factor is described by the size of its slice on the pie chart according to three levels of reactivity (low, medium, and high). **(A)** KIR2DL1 educated NK cells, KIR2DL2 educated NK cells, and KIR2DL3 educated NK cells present three educated NK profiles respectively associated with HLA-C1/C1, HLA-C1/C2, and HLA-C2/C2 genotypes. **(B)** KIR3DL1 educated NK cells present four educated NK profiles according to HLA-Bw4/Bw4, HLA-Bw4/Bw6, and HLA-Bw6/Bw6 genotypes. HLA-Bw4/BwX is distinguished by three different backgrounds: HLA-Bw4*80T, HLA-Bw4*80I with high expression of KIR3DL1, and HLA-Bw4*80I with low expression of KIR3DL1. **(C)** NKG2A educated NK cells present three educated NK profiles according to HLA-B-21M/M, HLA-B-21M/T, and HLA-B-21T/T genotypes. ND, no data is described. The ribbed area describes a superposition of two areas relative to the colors exhibited. The NK educated reactivity was adapted from *in vitro* HIV-infected CD4 cell stimulation assays, previously described (21, 72). Except for the Figure 2C, the NK educated IFNγ reactivity represented in these charts was taken *via in vitro* anti-CD20 Raji cell stimulation (74).

The magnitude of polyfunctionality (CCL4, CD107a, and IFN-γ) from KIR2DL2 educated NK cells (HLA-C1/C1) and KIR2DL1 educated NK cells (HLA-C2/C2) is the highest and most complete against HIV-infected cells. Conversely, KIR2DL1 educated NK cells (HLA-C1/C1), KIR2DL2 educated NK cells (HLA-C2/C2), and KIR2DL3 educated NK cells (HLA-C2/C2) are the effector educated NK cells with the most restricted polyfunctionality (21) (**Figure 2A**). High-density KIR3DL1 educated NK cells (HLA-Bw4*80I) showed a limited polyfunctionality equivalent to KIR2DL1/2/3 educated NK cells (HLA-C1/C2) (**Figures 2A, B**). Meanwhile, the low-density KIR3DL1 educated NK cells (HLA-Bw4*80I) and KIR3DL1 educated NK cells (HLA-Bw6) had a drastically restricted polyfunctionality (**Figure 2B**). Overall, the effector functions from KIR3DL1 educated NK cell subsets (any self-HLA-Bw4), excluding low-density KIR3DL1, uniquely favored CCL4 production against HIV-infected cells (21). Remarkably, high-density KIR3DL1 educated NK cells (HLA-Bw4*80I) were demonstrated to be more efficient at killing autologous HIV-infected CD4^+^ T cells than KIR3DL1 educated NK cells (HLA-Bw4*80T) (22), suggesting CCL4 could assist in NK cell-mediated HIV restriction.

As previously mentioned, NK cell education is not limited to KIR, and is also calibrated by NKG2A/HLA-E (8, 36). The HLA-B (-21M) genotype allows improved education of CD94/NKG2A^+^ NK cells over the HLA-B (-21T) genotype due to differences in the engagement of HLA-E (39). During HIV infection, NKG2A educated effector NK cell responses are also affected *via* HLA-B-21 polymorphisms. HLA-B-21M/M and HLA-A co-expression favors HLA-E expression, which in turn supports NKG2A educated NK cells. In persons living with HIV (PLWH) with HLA-B-21M/M profiles, a positive correlation between HLA-A expression and HIV viremia has been observed (75). On the contrary, no correlation between HLA-A expression and HIV viremia was observed in the HLA-B-21T/T genotype (75). Furthermore, NKG2A educated NK cells in individuals expressing HLA-B-21T/T showed higher degranulation and viral restriction against HIV-infected cells than NKG2A educated NK cells in individuals expressing HLA-B-21M/X (75, 76). However, NKG2A educated NK cells (in HLA-B-21M/M) exhibited higher IFN-γ production than NKG2A educated NK cells (HLA-B-21M/T) following ADCC stimulation (**Figure 2C**). The effector NKG2A educated NK cells (HLA-B-21M/M) seem to acquire an inflammatory profile by IFN-γ production with ADCC stimulation (74).

Overall, this data supports our proposition that effector educated NK cell responses are dependent on different environmental factors in the context of HIV infection, such as self-HLA-I profiles or anti-HIV antibody stimulation. According to these factors, the effector educated NK cell responses can induce vast changes in NK cell responses against target cells.

## Cytomegalovirus

**Figure 3:**
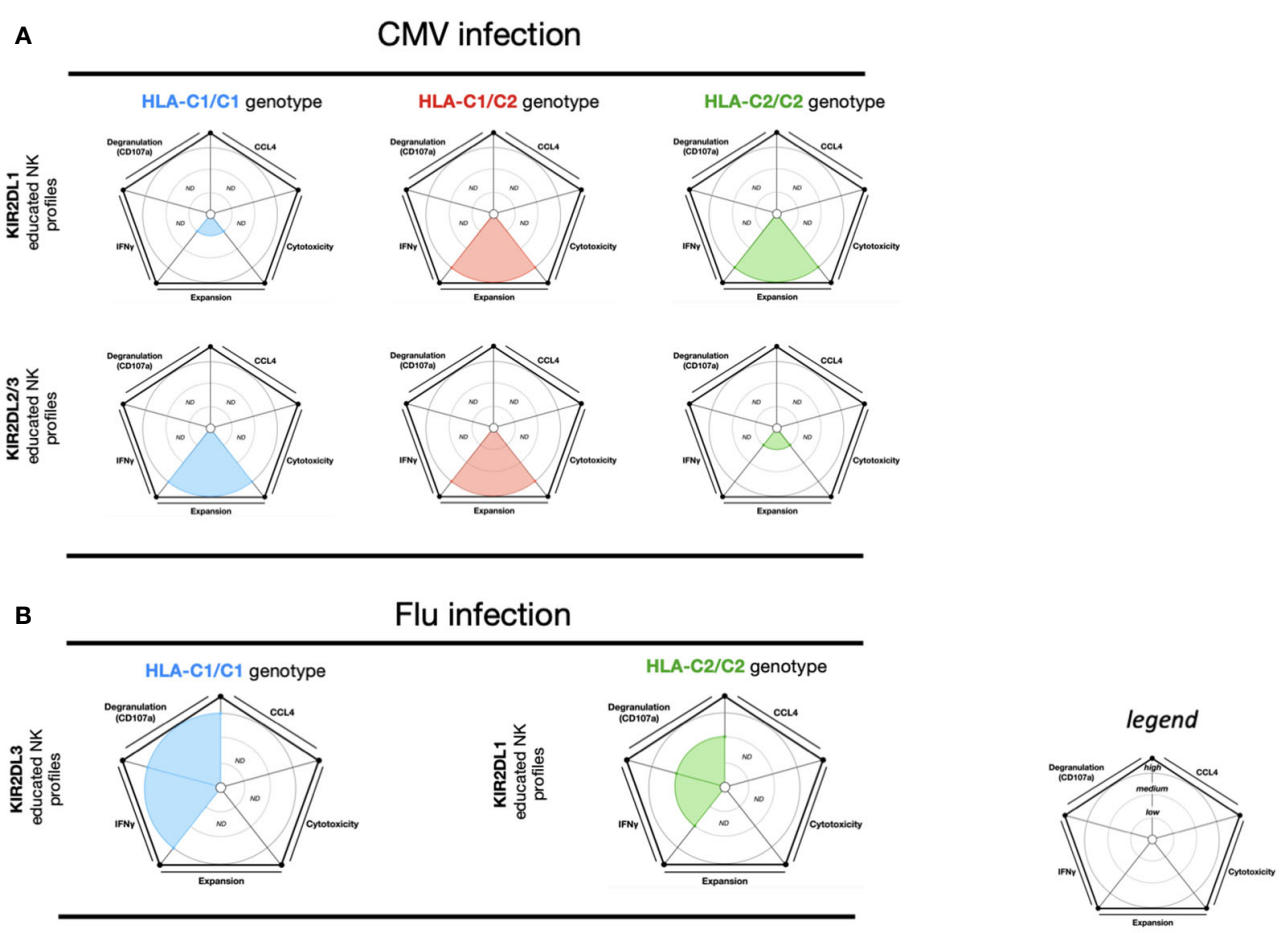
NK cell education profiles related to CMV and Influenza viral infections. The different colors each represent a specific-NKR educated NK profile. Each educated NK profiles are defined by five factors (degranulation, CCL4, IFNγ, cytotoxicity, and expansion). Each factor is described by the size of its slice on the pie chart according to three levels of reactivity (low, medium, and high). **(A)** KIR2DL1 educated NK cells and KIR2DL2/3 educated NK cells present respectively three educated NK profiles according to HLA-C1/C1, HLA-C1/C2, and HLA-C2/C2 genotypes. The NK educated reactivity represented in these charts was adapted *via in vitro* CMV-infected fibroblast stimulation (78, 81). **(B)** KIR2DL1 educated NK cells and KIR2DL3 educated NK cells each present a distinct educated NK profile according to the HLA-C1/C1 and HLA-C2/C2 genotypes, respectively. The NK educated reactivity represented in these charts was adapted *via in vitro* influenza A infected CD3-depleted PBMC cells stimulation (82). ND, no data is described.

NK cells play a critical role in the immune response to human CMV viral infection (77, 78). In particular, virus-induced NK cell clonal expansion and long-term persistence of “memory-like” NKG2C^+^CD57^+^self-KIR^+^ educated NK cells have a significant role in CMV infection (77–80). Within HLA-C1/C2 and HLA-C2/C2 genotypes from CMV-seropositive donors, KIR2DL1 educated NK cells expand upon coculture with CMV-infected fibroblasts (78, 81). Similarly, in HLA-C1/C1 and HLA-C1/C2 genotypes from CMV-seropositive donors, KIR2DL2 and KIR2DL3 educated NK cells also expand (78, 81). These data suggest that the proliferation of KIR educated NK cells by CMV-infected cell stimulation is relative to the HLA-I engaged during the NK cell education process (**Figure 3A**).

Overall, both KIR2DL1 educated NK cells and KIR2DL2/3 educated NK cells expand during human CMV infection (**Figure 3A**). This expansion is dependent on the association between educated NK cell and self-HLA-I, highlighting the crucial role of NK cell education in effector NK cell responses.

## Influenza virus

During the course of influenza infection, NK cells have been shown to provide an overall protective response (83). Using infected cell stimulation, one study demonstrated that KIR2DL1^+^ NK cells (HLA-C2/C2) showed lower IFN-γ and CD107a NK cell responses than KIR2DL3 NK cells (HLA-C1/C1) (82) (**Figure 3B**). These data suggest that the effector educated NK cell response from KIR2DL3 NK cells is more effective. Despite these findings, NK cell cytotoxic activity from the HLA-C1/C1 and HLA-C2/C2 groups did not differ against MHC-I negative target cells (82). In murine models of influenza infection, Ly49C/I uneducated NK cells proliferate more than Ly49C/I educated NK cells. Furthermore, Ly49-deficient and MHC-I-deficient murine models demonstrated that Ly49 uneducated NK cells are necessary for protection against influenza infection, specifically due to their cytotoxic functions (84).

## Dengue virus

Interestingly, NK cell responses from dengue-infected individuals were found to be independent of their education status. Indeed, iKIR (KIR2DL1, KIR2DL2, KIR2DL3, and KIR3DL1) educated NK cells and NKG2A educated NK cells from a pool of broad genotypic profiles (HLA-C1/C1, HLA-C1/C2, HLA-C2/C2, HLA-Bw4/Bw4, HLA-Bw4/Bw6, and HLA-Bw6/Bw6) did not show any differences in NK cell expansion between the educated and uneducated NK cell populations in acute dengue infection (85). However, the mixed pool of different educated NK cells and genotypes in this study could have reduced the potential impact of educated or uneducated NK cell activity during infection. Nonetheless, these analyses indicate that effector educated NK cell functions can be different according to the pathogen involved and provide a unique example where NK cell education may be less important (**Figures 2**, **3**).

## iNKR and genotype association: A fragment of NK cell education in viral infections

As previously explained, NK cell education is defined by the relationship between the self-HLA-I host background and iNKR expression which calibrate the activating threshold from effector NK cells. However, some studies have described the iNKR NK cell evolution in viral infection is associated with a specific genotypic profile, but without any functional NK cell description. Nevertheless, understanding this relationship could serve to develop our knowledge about the potential role of effector educated NK cell functions during other viral infections. For example, the combination of HLA-C1/C1 alleles and KIR2DL3 is associated with the resolution of hepatitis C virus (HCV) infection (86). In a study of severe acute respiratory syndrome coronavirus 2 (SARS-CoV-2) infection, an increase in KIR2DL1 and KIR2DL3 expression on NK cells was reported among the infected group compared to the non-infected healthy group (87). However, KIR2DL1 expression on NK cells was lower in severe disease compared to moderate disease (87). Furthermore, the number of individuals exhibiting a KIR2DL2 and HLA-C1/C1 association was lower in the severe symptom group than the asymptomatic group (88). Of note, the authors did not report any significant differences between SARS-CoV-2-infected patients and the healthy group with respect to HLA-C1/C1, HLA-C2/C2, HLA-C1/C2, HLA-Bw4I, and HLA-Bw4T genotypes (88). In Lassa virus (LASV) infection, NK cell activation can play an important role in the clearance of infected cells (89), except in individuals positive for both HLA-C1 and KIR2DL2, which have been associated with a significant increase in LASV replication and contribute to a fatal outcome in LASV infection. However, there were significant associations linked to protection in LASV infection between the HLA-C2 and KIR2DL1, HLA-C1 and KIR2DL3, or HLA-Bw4 and KIR3DL1 groups compared to the healthy control group (90). During acute chikungunya virus (CHIKV) infection, NK cells are activated with an increased frequency in the blood (91). One study has reported an enrichment of HLA-C1/C1 and HLA-C1/C2 genotypes in acute CHIKV-infected patients. These patients also expressed high levels of KIR2DL2/3 and low levels of KIR2DL1 on NK cells (92). Remarkably, a negative correlation between viral load and both KIR2DL1 and NKG2A expression on NK cells was also observed. Conversely, the authors found a positive correlation between viral load and KIR2DL2/3 expression on NK cells (92). While in some viral infections, specific KIR/HLA combinations are associated with control, the same combinations may facilitate fatal progression in others. Thus, these findings support our previous observations that effector educated NK cell functions are dependent on viral infections.

## NK cell education in tumor biology and transplantation

Tumors, like viruses, have evolved immune escape strategies *via* HLA downregulation. In this context, effector educated NK cells can potentially produce a strong NK cell response against MHC-I-deficient tumor cells. However, educated NK cells can also be inhibited against HLA-I-competent tumor cells *via* inhibitory ligand binding with poor activating signal interaction (93, 94). In addition, in a tumor context, NK cell education may also be led by TIGIT (43, 44), CD226 and SLAM (45, 46), as well as LIR-1 (LILRB1) (40) in addition to traditional KIR- and HLA-based education.

**Figure 4:**
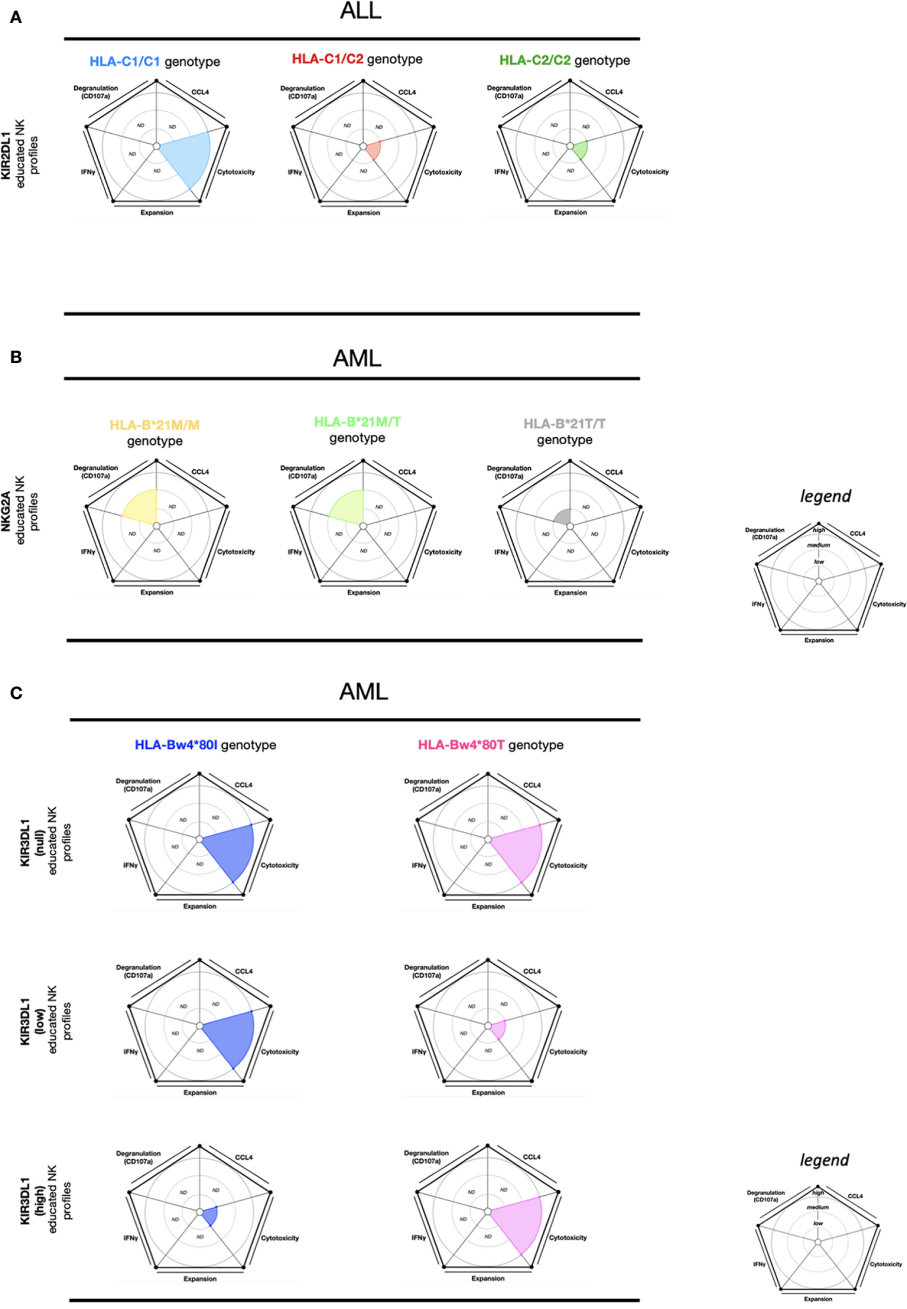
NK cell education profiles related to ALL and AML tumors. The different colors each represent a specific-NKR educated NK profile. Each educated NK profiles are defined by five factors (degranulation, CCL4, IFNγ, cytotoxicity, and expansion). Each factor is described by the size of its slice on the pie chart according to three levels of reactivity (low, medium, high). **(A)** KIR2DL1 educated NK cells present three educated NK profiles according to HLA-C1/C1, HLA-C1/C2, and HLA-C2/C2 genotypes. The NK educated reactivity represented in these charts was adapted *via in vitro* ALL blast stimulation (96). **(B)** NKG2A educated NK cells present three educated NK profiles according to HLA-B-21M/M, HLA- B-21M/T, and HLA- B-21T/T genotypes. The NK educated reactivity represented in these charts was adapted *via in vitro* AML blasts with IL- 2 stimulation (97). **(C)** KIR3DL1 educated NK cells present six educated NK profiles according to HLA-Bw4*80I and HLA-Bw4*80T genotypes and associated with the expression of KIR3DL1 (null, low, and high). The NK educated reactivity represented in these charts was adapted *via in vitro* AML cell line stimulation (98). ND, no data is described.

Like viral infections, HLA-I downregulation on acute lymphoblastic leukemia (ALL) blasts also contributes to escape from NK cell responses (95). Interestingly, upon *in vitro* stimulation with ALL cells, KIR2DL1 educated NK cells (HLA-C1/C1) show a higher cytotoxic activity than KIR2DL1 educated NK cells from HLA-C1/C2 and HLA-C2/C2 (96) (**Figure 4A**). Additionally, KIR2DL1 educated NK cells (HLA-C1/C1) also display higher cytotoxic activity than KIR2DL1 educated NK cells (HLA-C2/C2) and KIR2DL3 educated NK cells (HLA-C1/C1, HLA-C2/C2) upon *in vitro* stimulation with phytohemagglutinin (PHA). However, KIR2DL3 educated NK cells (HLA-C2/C2) exhibit a higher cytotoxic activity than KIR2DL1 educated NK cells (HLA-C2/C2) and KIR2DL3 educated NK cells (HLA-C1/C1) (96). This data suggests that effector KIR2DL1 educated NK cells (HLA-C1/C1) seem to be more efficient at killing ALL cells or PHA-blasts compared to KIR2DL1 and KIR2DL3 educated NK cells in most self-HLA-I profiles.

In acute myeloid leukemia (AML), HLA-E expression is downregulated in patients compared to healthy donors. Conversely, NKG2A is highly expressed on NK cells from AML patients, suggesting that AML patients might preserve inhibitory signals in NK cells through the NKG2A/HLA-E pathway (97). In HLA-B-21M/X (HLA-B-21M/M and HLAB-21M/T) genotypes and upon *in vitro* AML blast stimulation supplemented with IL- 2, NKG2A educated NK cells presented a higher CD107a expression compared to NKG2A educated NK cells from HLA-B-21T/T (**Figure 4B**). Moreover, iKIR educated NK cells from the HLA-B-21M/X and HLA-B-21T/T genotypes displayed lower CD107a expression than NKG2A educated NK cells (HLA-B-21M/X) (97). Supporting the findings of these *in vitro* assays, the overall survival among AML patients in IL- 2 treatment combined with histamine dihydrochloride (a drug administered to prevent relapse) was higher in the HLA-B-21M/X genotype group than in the HLA-B-21T/T genotype group (97). These data indicates that NKG2A educated NK cells display the most potent responses against AML blast cells in HLA-B-21M/X. Interestingly, HLA-B-21M promotes NKG2A NK cell education by a higher stabilization of HLA-E (39). Thus, the downregulation of HLA-E in AML patients can favor the activation of effector NKG2A educated NK cells. Overall, this data indicates that the threshold of activation from effector NKG2A educated NK cells is influenced by the HLA-B-21M/X genotype but also by an inflammatory environment *via* IL- 2 and supplementary activation signals.

Following *in vitro* AML cell line (SET-2 and KG-1) stimulation, KIR3DL1^low^ educated NK subsets and KIR3DL1 uneducated (KIR3DL1^null^) NK cells in the HLA-Bw4*80I genotype demonstrated high cytotoxic activity (98). On the contrary, the KIR3DL1^high^ educated NK cell subsets, as well as the KIR3DL1^null^ NK cells from the HLA-Bw4*80T genotype demonstrated high cytotoxic activity (98) (**Figure 4C**). In summary, these results show that effector KIR3DL1 educated NK cell function is efficient against AML in individuals expressing the following combinations: KIR3DL1^null^ and HLA-Bw4I/T, KIR3DL1^low^ and HLA-Bw4I, or KIR3DL1^high^ and HLA-Bw4T. These findings provide evidence that effector KIR3DL1^null^ NK cells provide the most efficient cytotoxic activity against AML in the HLA-Bw4 genotype profile.

Hematopoietic stem cell transplantation (HSCT) is a treatment strategy against myeloid malignant disease that has considerable anti-leukemic post-remission potential. However, relapse remains the main cause of mortality after HSCT (99). Effector educated NK cells from a donor (D^n^) play a key role in immune response reactivity and in the risk of relapse following HSCT (57, 100–102). Indeed, the educated NK cells must be calibrated by self-HLA-I molecules from a receiver (R^r^) to preserve immune tolerance after transplantation and reconstitute the effector NK cell response to conserve leukemia control.

**Figure 5:**
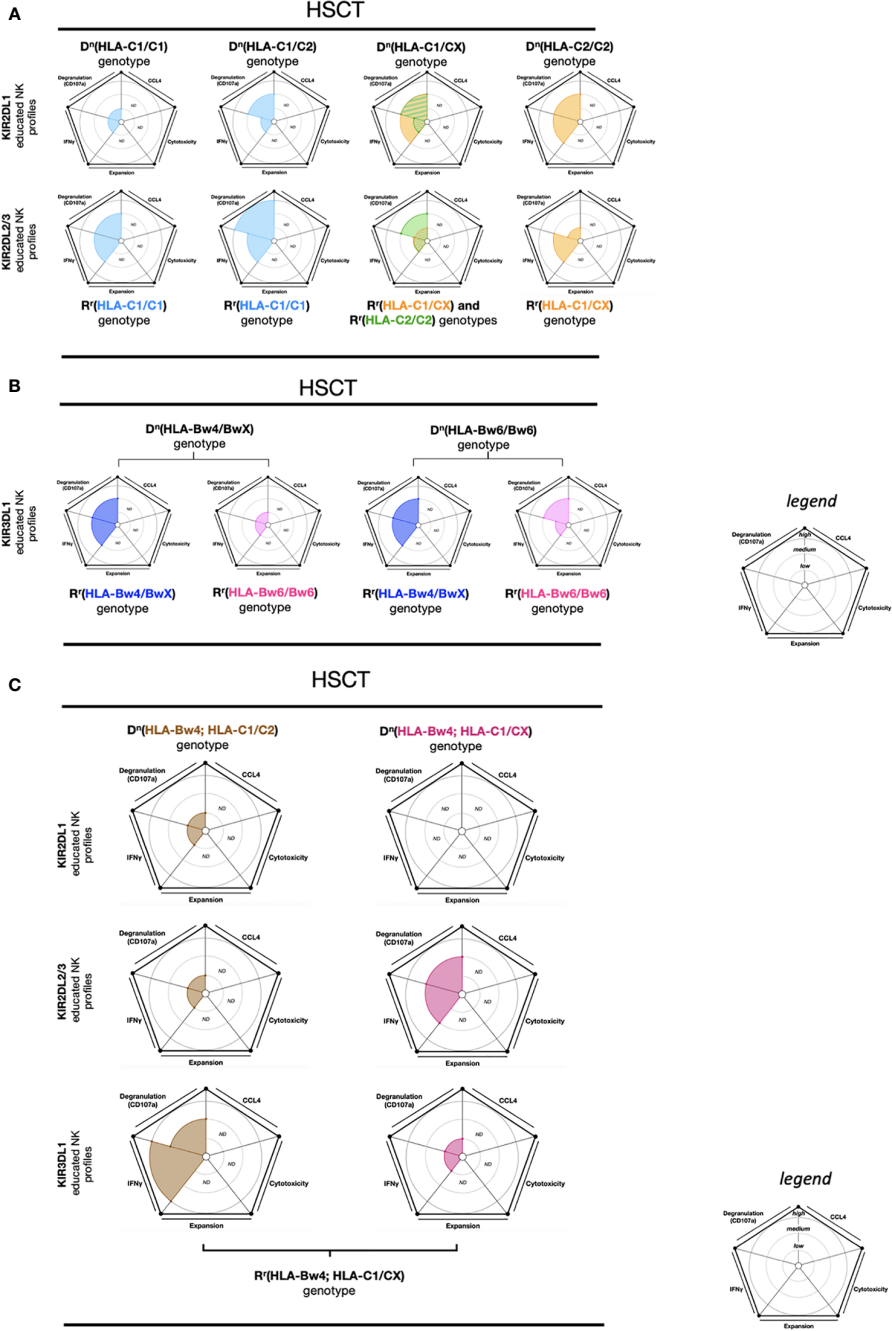
NK cell education profiles concerning to transplantation (HSCT). The different colors each represent a specific-NKR educated NK profile. Each educated NK profiles are defined by five factors (degranulation, CCL4, IFNγ, cytotoxicity, and expansion). Each factor is described by the size of its slice on the pie chart according to three levels of reactivity (low, medium, and high). **(A)** KIR2DL1 and KIR2DL2/3 educated NK cells present five educated NK profiles according to D^n^(HLA-C1/C1), D^n^(HLA-C1/C2), D^n^(HLA-C1/CX), and D^n^(HLA-C2/C2) genotypes as well as R^r^(genotype) associations. The NK educated reactivity represented in these charts was adapted *via in vitro* K562 cell stimulation (103). **(B)** KIR3DL1 educated NK cells present four educated NK profiles according to D^n^(HLA-Bw4/BwX) and D^n^(HLA-Bw6/Bw6) genotypes as well as R^r^(HLA-Bw4/BwX) or R^r^(HLA-Bw6/Bw6) associations. The NK educated reactivity represented in these charts was adapted *via in vitro* K562 cell stimulation (103). **(C)** KIR2DL1, KIR2DL2/3 and KIR3DL1 educated NK cells present six educated NK profiles according to D^n^(HLA-Bw4; HLA-C1/C2) and D^n^(HLA-Bw4; HLA-C1/CX) genotypes, associated with R^r^(HLA-Bw4; HLA-C1/CX). The NK educated reactivity represented in these charts was adapted *via in vitro* K562 cell stimulation (103). ND, no data is described. The ribbed area describes a superposition of two areas relative to the colors exhibited. The donor genotypes are denoted as D^n^ and receiver genotypes are denoted as R^r^.

Here we report the results of a study that measured effector educated NK cell reactivity 180 days post-HSCT to *in vitro* K562 cell stimulation (103). The effector KIR2DL1 educated NK cells in D^n^(HLA-C1/C1)-R^r^(HLA-C1/C1) and KIR2DL2/3 educated NK cells in D^n^(HLA-C1/CX)-R^r^(HLA-C1/CX) exhibited a low IFN-γ and CD107a response. Conversely, the effector KIR2DL1 educated NK cells in D^n^(HLA-C1/CX)-R^r^(HLA-C1/CX) and in D^n^(HLA-C2/C2)-R^r^(HLA-C1/CX) exhibited a dominant IFN-γ and CD107a response (103). Similarly, the effector KIR2DL2/3 educated NK cells in D^n^(HLA-C1/C1)-R^r^(HLA-C1/C1) and in D^n^(HLA-C1/C2)-R^r^(HLA-C1/C1) demonstrated a robust IFN-γ and CD107a response (**Figure 5A**). Therefore, the effector educated KIR2DL1 and KIR2DL2/3 NK cells present an adaptation to the R^r^ genotype as illustrated by the measure of high NK reactivity against cell line stimulation. The highest NK cell functional reconstitution for educated KIR2DL1 NK cells was observed in the context of D^n^(HLA-C1/CX and HLA-C2/C2)-R^r^(HLA-C1/CX) genotype associations. Additionally, the educated KIR2DL2/3 NK cells displayed functional reconstitution in the D^n^(HLA-C1/C1 and HLA-C1/C2)-R^r^(HLA-C1/C1) genotype associations.

KIR3DL1 educated NK cells also demonstrated great functional reconstitution, with a high IFN-γ and CD107a expression, in the D^n^(HLA-Bw4/BwX)-R^r^(HLA-Bw4/BwX) and D^n^(HLA-Bw6/Bw6)-R^r^(HLA-Bw4/BwX) genotype associations (103). In contrast, KIR3DL1 educated NK cells in D^n^(HLA-Bw4/BwX)-R^r^(HLA-Bw6/Bw6) revealed poor IFN-γ and CD107a expression (103) (**Figure 5B**). As expected, this data suggests that effector KIR3DL1 educated NK cells can recover their functions when exposed to HLA-Bw4.

Two multiple associations within the R^r^(HLA-Bw4; HLA-C1/CX) genotype, KIR3DL1 educated NK cells from D^n^(HLA-Bw4; HLA-C1/C2) and KIR2DL2/3 educated NK cells from D^n^(HLA-Bw4; HLA-C1/CX), reveal a functional reconstitution characterized by an important IFN-γ and CD107a expression against K562 cell stimulation (103). Conversely, KIR2DL1 educated NK cells from D^n^(HLA-Bw4; HLA-C1/C2), KIR2DL2/3 educated NK cells from D^n^(HLA-Bw4; HLA-C1/C2), and KIR3DL1 educated NK cells from D^n^(HLA-Bw4; HLA-C1/CX) display a deficient functional reconstitution illustrated by a low IFN-γ and CD107a expression against K562 cell stimulation (103) (**Figure 5C**).

Another study described a positive correlation between KIR expression and IFNγ production post-HSCT (3 months). Conversely, NKG2A educated NK cells reconstituted post-HCT (3 months) did not produce IFNγ despite the presence of degranulation markers (57). Moreover, it was suggested that the lower the number of KIR genes on the KIR haplotype, the more overall survival is improved, with each outcome conferring greater disease-free survival (104). To complete the KIR influences on the outcome of HSCT, a study demonstrated that reduced progression rate can also be dependent on the activating KIRs. The co-expression of KIR2DS2/3 and HLA-C1 can be strong for NK cell education and gradually weaker for KIR2DS1 and HLA-C2, and KIR3DS1 and HLA-Bw4 interactions, whereas the KIR2DS4 and HLA-A11/-C2/-C4/-C5/-C16 associations showed negligible impact (105). This mechanism can involve NK cell hyporesponsiveness to malignant cells in the receiver, deteriorate the overall survival, and accelerate the relapse rate in patients with malignancy after HSCT. In another study, a cohort of AML patients treated with autologous HSCT described a lower incidence of relapse due to the low-affinity interactions (KIR3DL1^+^ and HLA-Bw4-80T^+^, HLA-Bw4-80I^−^ genotypes) compared to a genotype with high-affinity interactions (KIR3DL1^+^ and HLA-Bw4-80I+ genotype) and this effect was also induced by a HLA-Bw4 copy number (106). These data suggest that high-affinity interactions confer strong inhibition, resulting in weak NK cell antileukemic activity in autologous HCT for AML patients.

In summary, the response of educated NK cells that occurs during transplantation is dependent upon donor and recipient genotypes. Indeed, the overall survival observed in HSCT exhibits a higher score in the D^n^(HLA-C1/C2,HLA-Bw4)-R^r^(HLA-C1/C2,HLA-Bw4) genotype than all other genotypes (103). However, the probability of relapse is higher in the D^n^(HLA-C1/C2) genotype with KIR3DL1/HLA-B strong inhibition than in the D^n^(HLA-C1/C2) genotype with KIR3DL1/HLA-B weak or non-inhibition (98). Thus, even if there is an HLA-I genotype match between the donor and recipient, unique multifactorial markers of educated NK cells may promote a significant increase in overall survival. Altogether, these results further suggest that the environment, while being specific to an individual, is important for effector educated NK cell reactivity, and the educated NK repertoire can adapt its response to another similar immune background.

## Conclusion

NK cells are essential to maintain healthy homeostasis and efficient immune responses against disease. In this review, we discussed how NK cell education is dependent upon a multitude of factors, such as iNKR variants, HLA-I expression variation, self-HLA-I peptide binding and soluble factors, among many others. All these features induce either effector NK hypo- or hyperresponsiveness by target cell recognition. However, neither the effector hypo- nor hyperresponsive NK cells are necessarily distinguished by educated or uneducated NK cells, respectively. Indeed, we put forward the idea that development of an adequate effector educated or uneducated NK cell response can fluctuate in response to different diseases. In this respect, other factors such as inflammatory or non-inflammatory soluble factor combinations can contribute to the identification of an optimal educated or uneducated NK cell response to specific diseases. The consideration of NK cell education is critical to promote a methodology favoring the resolution of pathologies *via* effector educated or uneducated NK cell responses and could aid in the development of NK cell-based immunotherapeutics.

## Author contributions

PR wrote the manuscript. PR, SJ and RR contributed to the conceptualization of the study. GW, SJ, CM, and RR edited and revised the manuscript. All authors contributed to the article and approved the submitted version.

## References

[1] Judge SJ Murphy WJ Canter RJ. Characterizing the dysfunctional NK cell: Assessing the clinical relevance of exhaustion, anergy, and senescence. Front Cell Infect Microbiol (2020) 10:49. doi: 10.3389/fcimb.2020.00049 32117816 PMC7031155

[2] Dogra P Rancan C Ma W Toth M Senda T Carpenter DJ. Tissue determinants of human NK cell development, function, and residence. Cell (2020) 180:749–763.e13. doi: 10.1016/j.cell.2020.01.022 32059780 PMC7194029

[3] Stokic-Trtica V Diefenbach A Klose CSN. NK cell development in times of innate lymphoid cell diversity. Front Immunol (2020) 11:813. doi: 10.3389/fimmu.2020.00813 32733432 PMC7360798

[4] Stary V Stary G. NK cell-mediated recall responses: Memory-like, adaptive, or antigen-specific? Front Cell Infect Microbiol (2020) 10. doi: 10.3389/fcimb.2020.00208 PMC7240046 32477964

[5] Kuroki K Furukawa A Maenaka K. Molecular recognition of paired receptors in the immune system. Front Microbiol (2012) 3. doi: 10.3389/fmicb.2012.00429 PMC3533184 23293633

[6] Morvan MG Lanier LL. NK cells and cancer: you can teach innate cells new tricks. Nat Rev Cancer (2016) 16:7–19. doi: 10.1038/nrc.2015.5 26694935

[7] Barrow AD Martin CJ Colonna M. The natural cytotoxicity receptors in health and disease. Front Immunol (2019) 10:909. doi: 10.3389/fimmu.2019.00909 31134055 PMC6514059

[8] Boudreau JE Hsu KC. Natural killer cell education and the response to infection and cancer therapy: Stay tuned. Trends Immunol (2018) 39:222–39. doi: 10.1016/j.it.2017.12.001 PMC6013060 29397297

[9] Orr MT Lanier LL. Natural killer cell education and tolerance. Cell (2010) 142:847–56. doi: 10.1016/j.cell.2010.08.031 PMC2945212 20850008

[10] Glasner A Oiknine-Djian E Weisblum Y Diab M Panet A Wolf DG. Zika virus escapes NK cell detection by upregulating major histocompatibility complex class I molecules. J Virol (2017) 91:e00623–17. doi: 10.1128/JVI.00785-17 28878071 PMC5660495

[11] Boudreau JE Hsu KC. Natural killer cell education in human health and disease. Curr Opin Immunol (2018) 50:102–11. doi: 10.1016/j.coi.2017.11.003 PMC5958620 29413815

[12] Highton AJ Diercks BP Möckl F Martrus G Sauter J Schmidt AH. High metabolic function and resilience of NKG2A-educated NK cells. Front Immunol (2020) 11:559576. doi: 10.3389/fimmu.2020.559576 33101277 PMC7554334

[13] Carrillo-Bustamante P de Boer RJ Keşmir C. Specificity of inhibitory KIRs enables NK cells to detect changes in an altered peptide environment. Immunogenetics (2018) 70:87–97. doi: 10.1007/s00251-017-1019-1 28695292 PMC5775373

[14] O’Connor GM Vivian JP Widjaja JM Bridgeman JS Gostick E Lafont BAP. Mutational and structural analysis of KIR3DL1 reveals a lineage-defining allotypic dimorphism that impacts both HLA and peptide sensitivity. J Immunol Baltim Md 1950 (2014) 192:2875–84. doi: 10.4049/jimmunol.1303142 PMC3948114 24563253

[15] Tu MM Mahmoud AB Makrigiannis AP. Licensed and unlicensed NK cells: Differential roles in cancer and viral control. Front Immunol (2016) 7. doi: 10.3389/fimmu.2016.00166 PMC4852173 27199990

[16] Brodin P Kärre K Höglund P. NK cell education: not an on-off switch but a tunable rheostat. Trends Immunol (2009) 30:143–9. doi: 10.1016/j.it.2009.01.006 19282243

[17] Goodson-Gregg FJ Krepel SA Anderson SK. Tuning of human NK cells by endogenous HLA-c expression. Immunogenetics (2020) 72:205–15. doi: 10.1007/s00251-020-01161-x PMC7182622 32219494

[18] Björkström NK Riese P Heuts F Andersson S Fauriat C Ivarsson MA. Expression patterns of NKG2A, KIR, and CD57 define a process of CD56dim NK-cell differentiation uncoupled from NK-cell education. Blood (2010) 116:3853–64. doi: 10.1182/blood-2010-04-281675 20696944

[19] Pende D Falco M Vitale M Cantoni C Vitale C Munari E. Killer ig-like receptors (KIRs): Their role in NK cell modulation and developments leading to their clinical exploitation. Front Immunol (2019) 10:1179. doi: 10.3389/fimmu.2019.01179 31231370 PMC6558367

[20] Pugh J. *In vitro* education of human natural killer cells by KIR3DL1. Life Sci Alliance (2019) 2:e201900434. doi: 10.26508/lsa.201900434 31723004 PMC6856763

[21] Kiani Z Dupuy FP Bruneau J Lebouché B Retière C Geraghty DE. The education of NK cells determines their responsiveness to autologous HIV-infected CD4 T cells. J Virol (2019) 93(23):e01185–19. doi: 10.1128/JVI.01185-19 31511383 PMC6854491

[22] Boudreau JE Mulrooney TJ Le Luduec J-B Barker E Hsu KC. KIR3DL1 and HLA-b density and binding calibrate NK education and response to HIV. J Immunol (2016) 196:3398–410. doi: 10.4049/jimmunol.1502469 PMC4868784 26962229

[23] Lutz CT. Human leukocyte antigen Bw4 and Bw6 epitopes recognized by antibodies and natural killer cells. Curr Opin Organ Transplant (2014) 19:436–41. doi: 10.1097/MOT.0000000000000103 PMC5742561 24977435

[24] Kim S Sunwoo JB Yang L Choi T Song YJ French AR. HLA alleles determine differences in human natural killer cell responsiveness and potency. Proc Natl Acad Sci (2008) 105:3053–8. doi: 10.1073/pnas.0712229105 PMC2268583 18287063

[25] Saunders PM Vivian JP Baschuk N Beddoe T Widjaja J O’Connor GM. The interaction of KIR3DL1*001 with HLA class I molecules is dependent upon molecular microarchitecture within the Bw4 epitope. J Immunol (2015) 194:781–9. doi: 10.4049/jimmunol.1402542 PMC4282956 25480565

[26] van der Ploeg K Le Luduec JB Stevenson PA Park S Gooley TA Petersdorf EW. HLA-a alleles influencing NK cell function impact AML relapse following allogeneic hematopoietic cell transplantation. Blood Adv (2020) 4:4955–64. doi: 10.1182/bloodadvances.2020002086 PMC7556125 33049053

[27] Saunders PM Pymm P Pietra G Hughes VA Hitchen C O’Connor GM. Killer cell immunoglobulin-like receptor 3DL1 polymorphism defines distinct hierarchies of HLA class I recognition. J Exp Med (2016) 213:791–807. doi: 10.1084/jem.20152023 27045007 PMC4854737

[28] Hilton HG Parham P. Missing or altered self: human NK cell receptors that recognize HLA-c. Immunogenetics (2017) 69:567–79. doi: 10.1007/s00251-017-1001-y PMC5560170 28695291

[29] Bari R Thapa R Bao J Li Y Zheng J Leung W. KIR2DL2/2DL3-E35 alleles are functionally stronger than -Q35 alleles. Sci Rep (2016) 6:23689. doi: 10.1038/srep23689 27030405 PMC4814820

[30] Le Luduec J-B Boudreau JE Freiberg JC Hsu KC. Novel approach to cell surface discrimination between KIR2DL1 subtypes and KIR2DS1 identifies hierarchies in NK repertoire, education, and tolerance. Front Immunol (2019) 10:734. doi: 10.3389/fimmu.2019.00734 31024561 PMC6460669

[31] Walzer T. NK-cell education: KIR-s come into play. Blood (2010) 115:1110–1. doi: 10.1182/blood-2009-11-254953 20150418

[32] Fauriat C Ivarsson MA Ljunggren H-G Malmberg K-J Michaëlsson J. Education of human natural killer cells by activating killer cell immunoglobulin-like receptors. Blood (2010) 115:1166–74. doi: 10.1182/blood-2009-09-245746 19903900

[33] Fadda L Borhis G Ahmed P Cheent K Pageon SV Cazaly A. Peptide antagonism as a mechanism for NK cell activation. Proc Natl Acad Sci (2010) 107:10160–5. doi: 10.1073/pnas.0913745107 PMC2890497 20439706

[34] Borhis G Ahmed PS Mbiribindi B Naiyer MM Davis DM Purbhoo MA. A peptide antagonist disrupts NK cell inhibitory synapse formation. J Immunol (2013) 190:2924–30. doi: 10.4049/jimmunol.1201032 PMC3672982 23382564

[35] Illing PT Pymm P Croft NP Hilton HG Jojic V Han AS. HLA-B57 micropolymorphism defines the sequence and conformational breadth of the immunopeptidome. Nat Commun (2018) 9:4693. doi: 10.1038/s41467-018-07109-w 30410026 PMC6224591

[36] Zhang X Feng J Chen S Yang H Dong Z. Synergized regulation of NK cell education by NKG2A and specific Ly49 family members. Nat Commun (2019) 10:5010. doi: 10.1038/s41467-019-13032-5 31676749 PMC6825122

[37] Celik AA Kraemer T Huyton T Blasczyk R Bade-Döding C. The diversity of the HLA-e-restricted peptide repertoire explains the immunological impact of the Arg107Gly mismatch. Immunogenetics (2016) 68:29–41. doi: 10.1007/s00251-015-0880-z 26552660 PMC4701785

[38] Rodgers JR Cook RG. MHC class ib molecules bridge innate and acquired immunity. Nat Rev Immunol (2005) 5:459–71. doi: 10.1038/nri1635 15928678

[39] Horowitz A Djaoud Z Nemat-Gorgani N Blokhuis J Hilton HG Béziat V. Class I HLA haplotypes form two schools that educate NK cells in different ways. Sci Immunol (2016) 1:eaag1672–eaag1672. doi: 10.1126/sciimmunol.aag1672 27868107 PMC5110269

[40] Leijonhufvud C Reger R Segerberg F Theorell J Schlums H Bryceson YT. LIR-1 educates expanded human NK cells and defines a unique antitumor NK cell subset with potent antibody-dependent cellular cytotoxicity. Clin Transl Immunol (2021) 10:e1346. doi: 10.1002/cti2.1346 PMC8491220 34631057

[41] Meazza R Falco M Marcenaro S Loiacono F Canevali P Bellora F. Inhibitory 2B4 contributes to NK cell education and immunological derangements in XLP1 patients. Eur J Immunol (2017) 47:1051–61. doi: 10.1002/eji.201646885 28386908

[42] Pende D Meazza R Marcenaro S Aricò M Bottino C. 2B4 dysfunction in XLP1 NK cells: More than inability to control EBV infection. Clin Immunol Orlando Fla (2019) 204:31–6. doi: 10.1016/j.clim.2018.10.022 30391652

[43] Chauvin JM Ka M Pagliano O Menna C Ding Q DeBlasio R. IL15 stimulation with TIGIT blockade reverses CD155-mediated NK-cell dysfunction in melanoma. Clin Cancer Res (2020) 26:5520–33. doi: 10.1158/1078-0432.CCR-20-0575 PMC8045409 32591463

[44] He Y Peng H Sun R Wei H Ljunggren HG Yokoyama WM. Contribution of inhibitory receptor TIGIT to NK cell education. J Autoimmun (2017) 81:1–12. doi: 10.1016/j.jaut.2017.04.001 28438433

[45] Wagner AK Kadri N Snäll J Brodin P Gilfillan S Colonna M. Expression of CD226 is associated to but not required for NK cell education. Nat Commun (2017) 8:15627. doi: 10.1038/ncomms15627 28561023 PMC5460037

[46] Chen S Yang M Du J Li D Li Z Cai C. The self-specific activation receptor SLAM family is critical for NK cell education. Immunity (2016) 45:292–304. doi: 10.1016/j.immuni.2016.07.013 27521267

[47] Netea MG Schlitzer A Placek K Joosten LAB Schultze JL. Innate and adaptive immune memory: an evolutionary continuum in the host’s response to pathogens. Cell Host Microbe (2019) 25:13–26. doi: 10.1016/j.chom.2018.12.006 30629914

[48] Sun JC. Re-educating natural killer cells. J Exp Med (2010) 207:2049–52. doi: 10.1084/jem.20101748 PMC2947064 20876314

[49] Burt BM Plitas G Zhao Z Bamboat ZM Nguyen HM Dupont B. The lytic potential of human liver NK cells is restricted by their limited expression of inhibitory killer ig-like receptors. J Immunol (2009) 183:1789–96. doi: 10.4049/jimmunol.0900541 PMC3253491 19587011

[50] Sharkey AM Xiong S Kennedy PR Gardner L Farrell LE Chazara O. Tissue-specific education of decidual NK cells. J Immunol (2015) 195:3026–32. doi: 10.4049/jimmunol.1501229 PMC4574523 26320253

[51] Salnikova LE Khadzhieva MB Kolobkov DS Gracheva AS Kuzovlev AN Abilev SK. Cytokines mapping for tissue-specific expression, eQTLs and GWAS traits. Sci Rep (2020) 10:14740. doi: 10.1038/s41598-020-71018-6 32895400 PMC7477549

[52] Fang P Li X Dai J Cole L Camacho JA Zhang Y. Immune cell subset differentiation and tissue inflammation. J Hematol Oncol.J Hematol Oncol (2018) 11:97. doi: 10.1186/s13045-018-0637-x 30064449 PMC6069866

[53] Zitti B Bryceson YT. Natural killer cells in inflammation and autoimmunity. Cytokine Growth Factor Rev (2018) 42:37–46. doi: 10.1016/j.cytogfr.2018.08.001 30122459

[54] Van Elssen CHMJ Oth T Germeraad WTV Bos GMJ Vanderlocht J. Natural killer cells: the secret weapon in dendritic cell vaccination strategies. Clin Cancer Res Off J Am Assoc Cancer Res (2014) 20:1095–103. doi: 10.1158/1078-0432.CCR-13-2302 24590885

[55] Curran SA Romano E Kennedy MG Hsu KC Young JW. Phenotypic and functional activation of hyporesponsive KIR neg NKG2A neg human NK-cell precursors requires IL12p70 provided by Poly(I:C)-matured monocyte-derived dendritic cells. Cancer Immunol Res (2014) 2:1000–10. doi: 10.1158/2326-6066.CIR-14-0054-T PMC4185245 25023628

[56] Wu Y Tian Z Wei H. Developmental and functional control of natural killer cells by cytokines. Front Immunol (2017) 8. doi: 10.3389/fimmu.2017.00930 PMC5543290 28824650

[57] Foley B Cooley S Verneris MR Curtsinger J Luo X Waller EK. NK cell education after allogeneic transplantation: dissociation between recovery of cytokine-producing and cytotoxic functions. Blood (2011) 118:2784–92. doi: 10.1182/blood-2011-04-347070 PMC3172795 21757615

[58] Juelke K Killig M Thiel A Dong J Romagnani C. Education of hyporesponsive NK cells by cytokines. Eur J Immunol (2009) 39:2548–55. doi: 10.1002/eji.200939307 19701893

[59] Wu CY Zhang B Kim H Anderson SK Miller JS Cichocki F. Ascorbic acid promotes KIR demethylation during early NK cell differentiation. J Immunol (2020) 205:1513–23. doi: 10.4049/jimmunol.2000212 PMC7484163 32759296

[60] Schafer JR Salzillo TC Chakravarti N Kararoudi MN Trikha P Foltz JA. Education-dependent activation of glycolysis promotes the cytolytic potency of licensed human natural killer cells. J Allergy Clin Immunol (2019) 143:346–358.e6. doi: 10.1016/j.jaci.2018.06.047 30096390

[61] Pfeifer C Highton AJ Peine S Sauter J Schmidt AH Bunders MJ. Natural killer cell education is associated with a distinct glycolytic profile. Front Immunol (2018) 9:3020. doi: 10.3389/fimmu.2018.03020 30619362 PMC6305746

[62] Brandstadter JD Yang Y. Natural killer cell responses to viral infection. J Innate Immun (2011) 3:274–9. doi: 10.1159/000324176 PMC3128146 21411975

[63] Ma L Li Q Cai S Peng H Huyan T Yang H. The role of NK cells in fighting the virus infection and sepsis. Int J Med Sci (2021) 18:3236–48. doi: 10.7150/ijms.59898 PMC8364442 34400893

[64] Capriotti T. HIV/AIDS: An update for home healthcare clinicians. Home Healthc. Now (2018) 36:348–55. doi: 10.1097/NHH.0000000000000706 30383593

[65] Reeves RK Li H Jost S Blass E Li H Schafer JL. Antigen-specific NK cell memory in rhesus macaques. Nat Immunol (2015) 16:927–32. doi: 10.1038/ni.3227 PMC4545390 26193080

[66] Huot N Rascle P Petitdemange C Contreras V Stürzel CM Baquero E. SIV-induced terminally differentiated adaptive NK cells in lymph nodes associated with enhanced MHC-e restricted activity. Nat Commun (2021) 12:1282. doi: 10.1038/s41467-021-21402-1 33627642 PMC7904927

[67] Albrecht C Malzahn D Brameier M Hermes M Ansari AA Walter L. Progression to AIDS in SIV-infected rhesus macaques is associated with distinct KIR and MHC class I polymorphisms and NK cell dysfunction. Front Immunol (2014) 5. doi: 10.3389/fimmu.2014.00600 PMC4246914 25506344

[68] Flórez-Álvarez L Hernandez JC Zapata W. NK cells in HIV-1 infection: From basic science to vaccine strategies. Front Immunol (2018) 9. doi: 10.3389/fimmu.2018.02290 PMC6199347 30386329

[69] Ende Z Deymier MJ Claiborne DT Prince JL Mónaco DC Kilembe W. HLA class I downregulation by HIV-1 variants from subtype c transmission pairs. J Virol (2018) 92:e01633-17. doi: 10.1128/JVI.01633-17 29321314 PMC5972908

[70] van Stigt Thans T Akko JI Niehrs A Garcia-Beltran WF Richert L Stürzel CM. Primary HIV-1 strains use nef to downmodulate HLA-e surface expression. J Virol (2019) 93:e00719-19. doi: 10.1128/JVI.00719-19 31375574 PMC6798123

[71] Nattermann J Nischalke H Hofmeister V Kupfer B Ahlenstiel G Feldmann G. HIV-1 infection leads to increased HLA-e expression resulting in impaired function of natural killer cells. Antivir Ther (2005) 10:95–107. doi: 10.1177/135965350501000107 15751767

[72] Körner C Simoneau CR Schommers P Granoff M Ziegler M Hölzemer A. HIV-1-Mediated downmodulation of HLA-c impacts target cell recognition and antiviral activity of NK cells. Cell Host Microbe (2017) 22:111–119.e4. doi: 10.1016/j.chom.2017.06.008 28704647 PMC5565794

[73] Martin MP Qi Y Gao X Yamada E Martin JN Pereyra F. Innate partnership of HLA-b and KIR3DL1 subtypes against HIV-1. Nat Genet (2007) 39:733–40. doi: 10.1038/ng2035 PMC4135476 17496894

[74] Cubero EM Ogbe A Pedroza-Pacheco I Cohen MS Haynes BF Borrow P. Subordinate effect of -21M HLA-b dimorphism on NK cell repertoire diversity and function in HIV-1 infected individuals of African origin. Front Immunol (2020) 11:156. doi: 10.3389/fimmu.2020.00156 32132995 PMC7041644

[75] Ramsuran V Naranbhai V Horowitz A Qi Y Martin MP Yuki Y. Elevated HLA-a expression impairs HIV control through inhibition of NKG2A-expressing cells. Science (2018) 359:86–90. doi: 10.1126/science.aam8825 29302013 PMC5933048

[76] Merino AM Sabbaj S Easlick J Goepfert P Kaslow RA Tang J. Dimorphic HLA-b signal peptides differentially influence HLA-e- and natural killer cell-mediated cytolysis of HIV-1-infected target cells: HLA-b signal peptides and NK cell function. Clin Exp Immunol (2013) 174:414–23. doi: 10.1111/cei.12187 PMC3826307 23952339

[77] Foley B Cooley S Verneris MR Pitt M Curtsinger J Luo X. Cytomegalovirus reactivation after allogeneic transplantation promotes a lasting increase in educated NKG2C+ natural killer cells with potent function. Blood (2012) 119:2665–74. doi: 10.1182/blood-2011-10-386995 PMC3311280 22180440

[78] Béziat V Liu LL Malmberg JA Ivarsson MA Sohlberg E Björklund AT. NK cell responses to cytomegalovirus infection lead to stable imprints in the human KIR repertoire and involve activating KIRs. Blood (2013) 121:2678–88. doi: 10.1182/blood-2012-10-459545 PMC3617633 23325834

[79] Della Chiesa M De Maria A Muccio L Bozzano F Sivori S Moretta L. Human NK cells and herpesviruses: Mechanisms of recognition, response and adaptation. Front Microbiol (2019) 10. doi: 10.3389/fmicb.2019.02297 PMC6788305 31636622

[80] Vivier E Raulet DH Moretta A Caligiuri MA Zitvogel L Lanier LL. Innate or adaptive immunity? the example of natural killer cells. Science (2011) 331:44–9. doi: 10.1126/science.1198687 PMC3089969 21212348

[81] Charoudeh HN Terszowski G Czaja K Gonzalez A Schmitter K Stern M. Modulation of the natural killer cell KIR repertoire by cytomegalovirus infection: Innate immunity. Eur J Immunol (2013) 43:480–7. doi: 10.1002/eji.201242389 23161492

[82] Ahlenstiel G Martin MP Gao X Carrington M Rehermann B. Distinct KIR/HLA compound genotypes affect the kinetics of human antiviral natural killer cell responses. J Clin Invest (2008) JCI32400:1017–26. doi: 10.1172/JCI32400 PMC2214845 18246204

[83] Li T. Respiratory influenza virus infection induces memory-like liver NK cells in mice. J Immunol (2017) 198:1242–52. doi: 10.4049/jimmunol.1502186 28031334

[84] Mahmoud AB Tu MM Wight A Zein HS Rahim MMA Lee SH. Influenza virus targets class I MHC-educated NK cells for immunoevasion. PloS Pathog (2016) 12:e1005446. doi: 10.1371/journal.ppat.1006210 26928844 PMC4771720

[85] Zimmer CL Cornillet M Solà-Riera C Cheung KW Ivarsson MA Lim MQ. NK cells are activated and primed for skin-homing during acute dengue virus infection in humans. Nat Commun (2019) 10:3897. doi: 10.1038/s41467-019-11878-3 31467285 PMC6715742

[86] Khakoo SI. HLA and NK cell inhibitory receptor genes in resolving hepatitis c virus infection. Science (2004) 305:872–4. doi: 10.1126/science.1097670 15297676

[87] Maucourant C Filipovic I Ponzetta A Aleman S Cornillet M Hertwig L. Natural killer cell immunotypes related to COVID-19 disease severity. Sci Immunol 5 (2020) 5:eabd6832. doi: 10.1126/sciimmunol.abd6832 PMC7665314 32826343

[88] Littera R Chessa L Deidda S Angioni G Campagna M Lai S. Natural killer-cell immunoglobulin-like receptors trigger differences in immune response to SARS-CoV-2 infection. PloS One (2021) 16:e0255608. doi: 10.1371/journal.pone.0255608 34352002 PMC8341547

[89] Russier M Reynard S Tordo N Baize S. NK cells are strongly activated by lassa and mopeia virus-infected human macrophages *in vitro* but do not mediate virus suppression: Innate immunity. Eur J Immunol (2012) 42:1822–32. doi: 10.1002/eji.201142099 22585682

[90] Wauquier N Petitdemange C Tarantino N Maucourant C Coomber M Lungay V. HLA-c-restricted viral epitopes are associated with an escape mechanism from KIR2DL2+ NK cells in lassa virus infection. EBioMedicine (2019) 40:605–13. doi: 10.1016/j.ebiom.2019.01.048 PMC6413685 30711514

[91] Hoarau JJ Jaffar Bandjee MC Krejbich Trotot P Das T Li-Pat-Yuen G Dassa B. Persistent chronic inflammation and infection by chikungunya arthritogenic alphavirus in spite of a robust host immune response. J Immunol (2010) 184:5914–27. doi: 10.4049/jimmunol.0900255 20404278

[92] Petitdemange C Becquart P Wauquier N Béziat V Debré P Leroy EM. Unconventional repertoire profile is imprinted during acute chikungunya infection for natural killer cells polarization toward cytotoxicity. PloS Pathog (2011) 7:e1002268. doi: 10.1371/journal.ppat.1002268 21966274 PMC3178577

[93] Mahaweni NM Ehlers FAI Sarkar S Janssen JWH Tilanus MGJ Bos GMJ. NKG2A expression is not per se detrimental for the anti-multiple myeloma activity of activated natural killer cells in an *In vitro* system mimicking the tumor microenvironment. Front Immunol (2018) 9:1415. doi: 10.3389/fimmu.2018.01415 29988376 PMC6023990

[94] Mahaweni NM Ehlers FAI Bos GMJ Wieten L. Tuning natural killer cell anti-multiple myeloma reactivity by targeting inhibitory signaling *via* KIR and NKG2A. Front Immunol (2018) 9:2848. doi: 10.3389/fimmu.2018.02848 30564241 PMC6288976

[95] Godal R Bachanova V Gleason M McCullar V Yun GH Cooley S. Natural killer cell killing of acute myelogenous leukemia and acute lymphoblastic leukemia blasts by killer cell immunoglobulin-like receptor–negative natural killer cells after NKG2A and LIR-1 blockade. Biol Blood Marrow Transplant (2010) 16:612–21. doi: 10.1016/j.bbmt.2010.01.019 PMC2854246 20139023

[96] Liu LL Béziat V Oei VYS Pfefferle A Schaffer M Lehmann S. Ex vivo expanded adaptive NK cells effectively kill primary acute lymphoblastic leukemia cells. Cancer Immunol Res (2017) 5:654–65. doi: 10.1158/2326-6066.CIR-16-0296 28637877

[97] Hallner A Bernson E Hussein BA Ewald Sander F Brune M Aurelius J. The HLA-b –21 dimorphism impacts on NK cell education and clinical outcome of immunotherapy in acute myeloid leukemia. Blood (2019) 133:1479–88. doi: 10.1182/blood-2018-09-874990 PMC6440292 30647027

[98] Boudreau JE Giglio F Gooley TA Stevenson PA Le Luduec JB Shaffer BC. KIR3DL1 / HL a-b subtypes govern acute myelogenous leukemia relapse after hematopoietic cell transplantation. J Clin Oncol (2017) 35:2268–78. doi: 10.1200/JCO.2016.70.7059 PMC5501362 28520526

[99] Zeiser R Vago L. Mechanisms of immune escape after allogeneic hematopoietic cell transplantation. Blood (2019) 133:1290–7. doi: 10.1182/blood-2018-10-846824 30578254

[100] Parikh BA Bern MD Piersma SJ Yang L Beckman DL Poursine-Laurent J. Control of viral infection by natural killer cell inhibitory receptors. Cell Rep (2020) 32:107969. doi: 10.1016/j.celrep.2020.107969 32726632 PMC7458139

[101] Haas P Loiseau P Tamouza R Cayuela JM Henry G Falk CS. NK-cell education is shaped by donor HLA genotype after unrelated allogeneic hematopoietic stem cell transplantation. Blood (2011) 117:9. doi: 10.1182/blood-2010-02-269381 21045194

[102] Zhao Y Gao F Wu Y Shi J Luo Y Tan Y. Decreased iKIR-HLA c pair confers worse clinical outcomes for patients with myeloid disease receiving antithymocyte globulin-based haploidentical hematopoietic stem cell transplantation. Front Immunol (2021) 11:614488. doi: 10.3389/fimmu.2020.614488 33633734 PMC7901980

[103] Zhao XY Yu XX Xu ZL Cao XH Huo MR Zhao XS. Donor and host coexpressing KIR ligands promote NK education after allogeneic hematopoietic stem cell transplantation. Blood Adv (2019) 3:4312–25. doi: 10.1182/bloodadvances.2019000242 PMC6929384 31869417

[104] Escudero A Martínez-Romera I Fernández L Valentín J González-Vicent M Vicario JL. Donor KIR genotype impacts on clinical outcome after T cell-depleted HLA matched related allogeneic transplantation for high-risk pediatric leukemia patients. Biol Blood Marrow Transplant J Am Soc Blood Marrow Transplant (2018) 24:2493–500. doi: 10.1016/j.bbmt.2018.08.009 30145228

[105] Nowak J Kościńska K Mika-Witkowska R Rogatko-Koroś M Mizia S Jaskuła E. Role of donor activating KIR-HLA ligand-mediated NK cell education status in control of malignancy in hematopoietic cell transplant recipients. Biol Blood Marrow Transplant J Am Soc Blood Marrow Transplant (2015) 21:829–39. doi: 10.1016/j.bbmt.2015.01.018 25617806

[106] Marra J Greene J Hwang J Du J Damon L Martin T. KIR and HLA genotypes predictive of low-affinity interactions are associated with lower relapse in autologous hematopoietic cell transplantation for acute myeloid leukemia. J Immunol Baltim Md 1950 (2015) 194:4222–30. doi: 10.4049/jimmunol.1402124 25810393

